# Characterization of the complete mitochondrial genome of *Cerogria popularis* Borchmann, 1936 (Coleoptera: Tenebrionidae)

**DOI:** 10.1080/23802359.2022.2072247

**Published:** 2022-05-09

**Authors:** Tongtong Liu, Jiansheng Qiu, Yuekai Wu, Kai Hu

**Affiliations:** Guizhou Academy of Forestry, Guiyang, P. R. China

**Keywords:** Mitogenome, phylogenetic analysis, Lagriinae

## Abstract

In this study, we sequenced the complete mitochondrial genome (mitogenome) of *Cerogria popularis* Borchmann, 1936 based on the Illumina platform. The circular DNA molecule is 16,175 bp in size, including the 37 typical animal mitochondrial genes and a non-coding control region. All 37 genes are arranged in the same order as the previously reported most mitogenomes of Tenebrionidae. All PCGs initiate with standard start codon ATN (ATA/T/G/C) except *cox1* with the special start codon AAT. Most PCGs terminate with TAA/G, whereas *cox1*, *atp6*, *nad5*, and *nad4* end with its incomplete form T-. All the 22 tRNAs have the typical clover-leaf structure except for *trnS1*. The *rrnL* and *rrnS* genes are 1,250 and 737 bp in length, with an AT content of 82.6 and 84.5%, respectively. The phylogenetic tree supports the monophyly of the included tenebrionid subfamilies represented by more than one species. Furthermore, the sister relationship between Lagriinae and other tenebrionid subfamilies is recovered.

The subfamily Lagriinae belongs to the family Tenebrionidae of Coleoptera, and to date there are more than 2,200 species (subspecies) described worldwide (Zhou and Chen 2014). Its range and taxonomic status have been debated throughout the history of research (Zhu 2003). This study firstly sequenced the complete mitochondrial genome (mitogenome) of *Cerogria popularis* Borchmann, 1936, which will be useful for exploring the phylogenetic status of Lagriinae.

The samples of *C. popularis* were collected from Weining County (E104°36′39.63″, N26°48′44.16″), Guizhou Province, China and deposited in Insect Herbarium of Guizhou Academy of Forestry, Guiyang (GZAF-2021-CT0078) (URL, Kai Hu and 18792617323@163.com). Identification of adult specimens was based on morphological characteristics (Zhu 2003; Zhou 2011). The total genome DNA was sequenced using Illumina MiSeq format (Illumina, San Diego, CA). Then the data was assembled by NOVOPlasty v4.3.1 (Dierckxsens et al. 2017) with the *cox1* gene of *Chrysomela vigintipunctata* (NC_050933) as the seed. The complete mitogenome was annotated using MITOZ v1.04 (Meng et al. 2019). All 13 protein-coding gene sequences were aligned using MAFFT v7.394 (Kuraku et al. 2013) with L-INS-i (accurate) strategy. Maximum likelihood (ML) tree was inferred by IQ-TREE v1.6.3 (Nguyen et al. 2015) under the optimal model (GTR + I + G for Subset1 (*cox2*, *cytb*, *cox3*, *nad3*, and *atp6*), Subset2 (*nad2*, *nad6*, and *atp8*), Subset3 (*cox1*), and Subset4 (*nad1*, *nad4L*, *nad4*, and *nad5*)).

The mitogenome of *C. popularis* (GenBank accession no. MZ699994) is 16,175 bp in size, which comprises 13 protein-coding genes (PCGs), 22 transfer RNA genes (tRNAs), two ribosomal RNA genes (rRNAs), and a non-coding control region (length: 1,692 bp). All 37 genes are arranged in the same order as the previously sequenced most mitogenomes of Tenebrionidae (Hong et al. 2020). The overall nucleotide composition of the newly sequenced mitogenome is 41.9% T, 10.5% C, 38.3% A, and 9.3% G, with an AT content of 80.2%. All PCGs initiate with standard start codon ATN (ATA/T/G/C) except *cox1* with the special start codon AAT. Most PCGs terminate with TAA/G, whereas *cox1*, *atp6*, *nad5*, and *nad4* end with its incomplete form T-. All tRNAs exhibit a typical clover-leaf secondary structure except for *trnS1*, in which the dihydrouridine (DHU) arm is replaced by a simple loop. The *rrnL* and *rrnS* genes are 1,250 and 737 bp in length, with an AT content of 82.6 and 84.5%, respectively. The control region is located between *rrnS* and *trnI*, which is 1,692 bp in length.

In this study, based on the nucleotide data of 13 PCGs from 17 Tenebrionidae species and two outgroup taxa from Cerambycidae, we reconstructed the ML phylogenetic tree (Figure 1). The phylogenetic analyses strongly support the monophyly of the included tenebrionid subfamilies represented by more than one species (Alleculinae, Stenochiinae, Tenebrioninae, and Pimeliinae) (BS ≥95), similar to the recent study (Hong et al. 2020), but our taxon sample is much larger. In Tenebrionidae, the relationships among included subfamilies are inferred as (Lagriinae + (Pimeliinae + (Tenebrioninae + (Stenochiinae + (Alleculinae + Diaperinae))))). Furthermore, the sister relationship between Lagriinae and other tenebrionid subfamilies is recovered (BS = 100).

**Figure 1.**
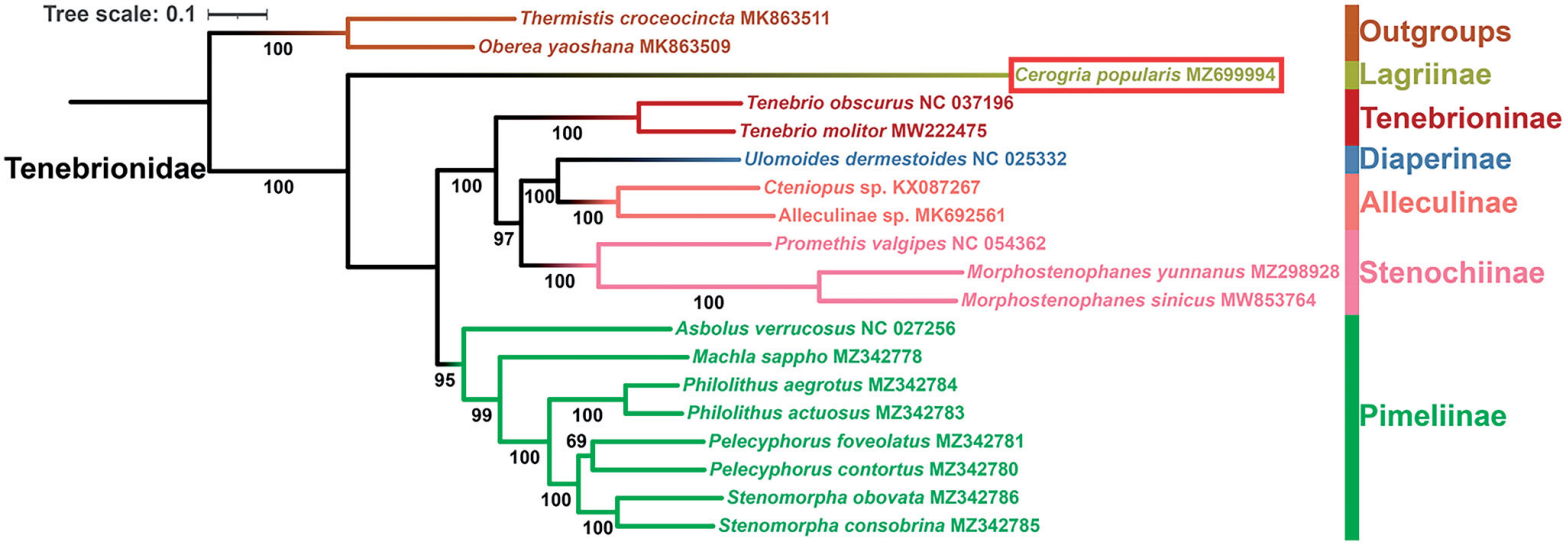
Maximum likelihood phylogenetic tree for Tenebrionidae based on the nucleotide sequence data of 13 protein-coding genes from *Cerogria popularis* and other 18 species belonging to seven subfamilies of Coleoptera. The number on each node indicates the bootstrap support value (BS).

## Data Availability

The genome sequence data that support the findings of this study are openly available in GenBank of NCBI at (https://www.ncbi.nlm.nih.gov/) under the accession no. MZ699994. The associated BioProject, SRA, and Bio-Sample numbers are PRJNA763012, SRR15890679, and SAMN21419663, respectively.
